# Observations on Aneurism, and Its Treatment by Compression

**Published:** 1847-09

**Authors:** 


### REVIEWS.

---

ART. V.—*Observations on Aneurism, and its Treatment by Compression.* By O'BRYEN BELLINGHAM, M.D., Edin.; Fellow of and Professor in the School of the Royal College of Surgeons in Ireland; Licentiate of the Royal College of Surgeons of Edinburgh, and one of the Surgeons to St. Vincent's Hospital. London: John Churchill. 1847. pp. 181. 12mo.

We have read this little volume with much pleasure. There is an air of candor, modesty, and good-faith pervading it, which in these days of dissimulation, arrogance, and deceit, is altogether agreeable. We like even the controversial portions of it, for which the author thinks it necessary to offer an apology; for, if the editor of the Dublin Quarterly Journal of Medicine has behaved as is here represented, he deserves all that he gets, and something more.

Dr. Bellingham, in the former chapters, traces succinctly, but clearly, the history of compression as applied to the cure of aneurisms. It seems to have had its origin in the application of compresses and bandages to wounded arteries, and was first applied to traumatic aneurism, especially that arising from accidental wound of the brachial artery in venesection. This was so frequent in the time of Dionis, that in France surgeons were compelled by law to pension those whose arms they had injured. Even at a more recent period in England John Bell inveighs against the bungling manner in which venesection was performed; "Masons, butchers, gardeners, cowherds! are among the chief phlebotomists in this country, and it is to them we are indebted for the various specimens of aneurism." Galen is said to have cured an aneurism at the bend of the arm by means of sponge and bandages. According to Sprengel, De Vigo first used compression, in the sixteenth century. Tulpius, writing in 1641, has recorded a case cured by this means; the aneurism occurred between the forefinger and thumb in consequence of punctured wound. Pilas reported a case of brachial aneurism in 1671; and Genga, in 1673, successfully treated a third case by compresses, and a bandage which took his name. But the Abbé Bourdelot was the first to invent and apply a compressing instrument for the cure of aneurism.

"This instrument, named a *Ponton*, appears to have been a very simple contrivance; it consisted merely of a pad of a peculiar shape, with leathern straps attached, which passed round the arm, above and below the elbow, and were perforated with holes to permit of their being tightened or loosened. The pad had a hollow or channel on its under surface, which it was supposed would permit the blood to pass freely down the artery under it, as a bridge (ponton) permits the water to flow under it, (hence the name)."

The aneurism occurred at the bend of the arm in his own person, in consequence of a puncture of the brachial artery, and was cured by wearing for a year the instrument described above. Other cases of traumatic aneurism of the brachial artery are reported a few years later by Rogerus and Rommelius; but these were treated by compresses and bandages. Besides the *ponton* of Bourdelot, an instrument invented by Scultetus was employed about this time.

"Scultetus's apparatus consisted of two flat metal hoops or rings, into which the arm readily fitted, they were joined by connecting flat pieces, into one of which a screw with a pad at its extremity was fixed; the instrument was sufficiently long to reach from the elbow to the wrist, and pressure was effected by turning the screw. Scultetus invented it for wounds of the palmar arch, but it came afterwards to be used in wounds or aneurism of the brachial artery."

Down to the middle of the seventeenth century compression, whether by instruments or bandages, was limited to recent cases of traumatic aneurism of the brachial artery, where the tumor was yet small, and the skin uninflamed. Pressure was supposed to act by forcing the blood from the tumor back into the artery, when the edges of the arterial opening came into contact and adhered; so that, the pressure being discontinued, the blood flowed along the artery as before.

The tourniquet was invented about the beginning of the eighteenth century, and a variety of instruments for the compression of aneurism made their appearance. Those of Vallant, Senffio, Arnaud, Heister, and Foubert, are briefly noticed by the author. The use of these instruments was still confined to recent and small traumatic aneurisms of the brachial artery and branches, or to small arteries in other parts of the body. When the tumor attained much volume, surgeons had recourse to the knife: the sac was laid open in its whole length, the coagula and fluid blood removed, and the artery tied above and below the aneurism; the wound was then filled with lint, and bandages and compresses applied.

About the middle of the eighteenth century Heister first proposed compression in popliteal aneurism, but he does not appear to have put it in practice. This honor belongs to Guattani, an Italian surgeon.

"He appears to have been induced to try this method, partly from the success which attended the employment of pressure in aneurism at the bend of the arm following venesection; and partly, from his having witnessed a spontaneous cure in three cases of popliteal aneurism; but principally in consequence of the great fatality of aneurism of this vessel, and the lamentable results of every operation for its relief. Popliteal aneurism is a disease (he observes) in which medicine holds out no prospect of cure, in which surgery has almost abandoned all treatment, and in which physicians and surgeons are agreed only in the prognosis."

In two cases of aneurism of the popliteal artery he had tried the operation usually performed for brachial aneurism; one of the patients was cured, the other died of gangrene. In four cases he had tried amputation of the limb above the seat of disease; but all terminated fatally, two dying of hemorrhage, and the remaining two of tetanus. He then resolved on methodical compression and bandages, combined with low diet, perfect rest, and occasional bleeding. We quote one of the cases as given by Dr. Bellingham; it will serve as a specimen of the whole.

"The patient, a porter, aged 40 years, was admitted into the hospital of the Holy Ghost towards the end of August, 1765, laboring under popliteal aneurism. The tumor was about the size of a large goose egg, was hard and resistant to the touch, had a strong pulsation, and was attended by pain, swelling of the leg and foot, and fever. The patient was directed to remain in bed, was put upon low diet, and bled several times. Towards the end of September the aneurism had ceased to increase, pain was nearly gone, the pulsation had much diminished, and the swelling of the limb had in a great measure subsided.

"Guattani was obliged to leave Rome at this period to accompany the Pope on a journey. On his return in November the tumor had not increased, and the œdema of the limb having disappeared, he determined to try the effects of compression. Having first covered the tumor with lint, he laid two oblong compresses over its centre, which crossed one another, and passed round the limb above and below the knee; another compress was laid along the course of the femoral artery up to the groin. He then took a long roller, three fingers' breadth, with which he encircled the knee, commencing above the centre of the tumor, and passing it above and below the joint many times; it was then carried up the thigh to the groin, and for further security he gave it a turn round the body. The whole was moistened with spirits of wine, the patient was bled, and a rigid diet enforced. The bandage was not disturbed for eighteen or twenty days, and then only in order to be tightened; and venesection was repeated whenever the leg or foot swelled.

"The tumor, although it remained hard and continued to pulsate, gradually decreased in size, and in three months from the first application of the compression, the patient left the hospital perfectly well. At this period the tumor scarcely exceeded the size of a bean; and although the patient was obliged to return to his former business, and was frequently obliged to lift heavy weights, he suffered no inconvenience except slight œdema of the leg and foot, which did not prevent him from following his occupation of a porter."

Guattani tried this method in crural and inguinal aneurism, but without success. Of popliteal aneurism he reports twenty cases—

—"three of these underwent a spontaneous cure; six were operated on, of which one recovered and five died, and eleven were treated by compression, of which four were perfectly cured and one so much benefited, that it is probable he ultimately recovered; while in six it failed. The statistics of Guattani's cases are, therefore, greatly in favor of compression in popliteal aneurism (as he practised it) over any operation then known; but the results of the treatment by laying open the sac and tying the artery above and below it were even less satisfactory in the hands of others at the period in question, and the danger which attended this operation led surgeons in England to abandon it, and to recommend amputation of the limb in preference."

Guattani's method, it will be seen, was merely the old one in use for brachial aneurism, combined with the medical treatment proposed by Valsalva and Albertini for internal aneurism. It was liable to several objections: the pressure might convert a circumscribed into a diffused aneurism; might lead to inflammation and suppuration of the sac, and to gangrene of the limb. It was painful from compression of the surrounding parts when the sac was large, but especially from stretching and compression of the large nerve which lies between the sac and integuments. It was tedious, owing to the necessity of frequently removing the bandages on account of pain, and the shrinking of the limb.

"Now, as this mode of treatment appears to have been seldom employed except in cases of aneurism of some standing in which more or less fibrin is always deposited in the interior of the sac, when the tumor is necessarily, therefore, more or less solid, and too resistant to be diminished much by pressure, its pulsation could only have been lessened or suspended by the sac being made to press upon the artery above, and perhaps in some instances also below it; and the cure of the aneurism must have been eventually accomplished by the obliteration of the vessel here. In such cases, the compression of the entire limb by tight bandages tended rather to retard than to accelerate this event, by interfering with the enlargement of the collateral vessels about the knee."

This method was employed by Flajani, Paletta, and Vicq d'Azyr, but the introduction of the Hunterian operation, a few years afterwards, set it aside. The case reported by Vicq d'Azyr was one of femoral aneurism, which he cured by a compressing instrument of his own invention.

"It is a modification of the tourniquet, the bridge in which the screw works has a pad attached, which is acted on by the latter; and a flat splint covered with leather is connected to it by strong bands by which counter-pressure is made. The pad is laid upon the aneurism and pressure is made by turning the screw, while the splint is applied to the opposite side of the limb. The screw is turned by a key, which can be removed when the proper amount of pressure is made, so that the patient cannot loosen it himself."

Another mode of applying pressure, called *immediate* to distinguish it from the preceding, was sometimes employed. It consisted in opening the sac and, after clearing away the coagula, laying graduated compresses on the vessel, where they were retained by bandages, rollers, &c.

"This mode of using compression had not, however, many advocates; because if very firm pressure was made, there was a risk of the artery being obliterated at the part; whereas if the pressure was moderate, secondary hemorrhage was very likely to occur. Indeed Murray relates two cases, where (owing to the latter cause) the patients who had been operated on for brachial aneurism according to this method, bled to death during sleep, from the bandages having become accidentally disturbed."

Hitherto, says the author, the names of but few British surgeons appear in connection with improvements in the treatment of aneurism, and none in connection with the treatment by compression. The continental surgeons had taken the lead, especially those of the French and Italian schools. But in 1785, Hunter performed his first operation for popliteal aneurism, and since then British surgery has mingled largely in the treatment of this disease. The simplicity of this operation, the facility with which it was performed, and the success attending it, caused it to supercede almost entirely the method of compression. Still, in some instances it failed, and, in others, owing to the situation of the aneurism, was impracticable. In these recourse was had to pressure on the cardiac side of the tumor. Pressure was thought to effect what the ligature effected in the Hunterian operation.

"The principal upon which surgeons applied pressure at this period was to endeavor to excite inflammation of the coats of the artery at the seat of its application: they supposed that if by means of strong pressure the coats of an artery were brought into contact, and so retained, inflammation of the arterial tunics would follow, lymph would be thrown out, adhesion would take place between them, and the obliteration of the artery would be effected in the same manner as if a ligature had been applied. The first to endeavor to determine by direct experiment the effects of pressure npon the arterial tissue, was Mr. Freer, of Birmingham, who, in the year 1806, performed a series of experiments upon animals with this object. He found that compression when applied with sufficient force to excite inflammation of the artery and the parts surrounding it, instead of causing lymph to be thrown out in the cavity of the artery, and adhesion of the sides of the vessel, (as was commonly supposed) occasioned an effusion of lymph about the artery and between its coats, by which its tube was so much compressed as to be impervious to the passage of blood."

Unsuccessful attempts with this mode of compression were made by several British surgeons. The earliest examples of its successful employment in popliteal aneurism are recorded by Pelletan, Dubois, and Viricel. Dupuytren and Boyer likewise report successful cases, but in others it failed in their hands. The first successful case among the Irish surgeons is that of Mr. McCoy, which occurred at Dublin in 1824. Todd, of the same city, a year or two previous, had tried compression as preparatory to ligation of the artery, and had invented for the purpose an instrument resembling a truss for femoral hernia. He also employed compression with success for the cure of aneurism in 1826, but the case was not made public until 1846. Another case occurred in the practice of Verdier on the continent; this was similar in many respects to that reported by McCoy. From this period to the year 1843, the author finds but few instances where compression was tried. One reason assigned for this is the increased confidence surgeons seem to have reposed in the ligature. Another reason is thus hinted at:

"Perhaps the *eclat* to be gained by the successful performance of a capital operation was not without its influence in inducing surgeons to give the preference to a mode of treating aneurism, which afforded the opportunity for this display, over the quieter and less imposing proceeding by compression; particularly as the former had received the sanction of long experience, and was supposed to be the only certain method of curing aneurism."

In the meantime, as with the old method of compression, so with the new, a modification termed *immediate* was proposed, in which, after cutting down upon the artery, above the sac, as if to apply the ligature, pressure is applied directly to the vessel. This modification was suggested very soon after the introduction of the Hunterian operation. The design was to cause obliteration of the artery without previous division of its coats, hoping thus to diminish the chances of secondary hemorrhage. Hence the recommendation of broad ligatures by the surgeons of that day, with cylinders of linen, pieces of cork, wood, leather, or agaric, and sometimes a portion of bougie, placed lengthwise between the vessel and the ligature. These means soon gave place to a great variety of instruments, which it was supposed would have considerable advantage over the ligature, as the pressure could be regulated at will, and could be removed altogether when a sufficient degree of inflammation had been excited in the arterial coats. Deschamps led the way in 1796 with an instrument, and was soon followed by Ayrer, Percy, Dubois, Assalini, Crampton, and others, with all sorts of *presse-artères*, or *serre-artères.* Some cures were effected in this way, but the general success was by no means encouraging.

It was also proposed to make pressure on the distal side of the aneurism, corresponding to Brasdor's modification of the Hunterian operation. This plan, in the few trials made of it, was found rather to aggravate the symptoms. Our author tried it "for a short time in a case of secondary iliac aneurism where compression could not be applied above the sac, where it was probable the blood entered the sac in a retrograde course through the distal end of the vessel, and where an operation of course was out of the question." Cure was afterwards brought about by direct pressure upon the sac.

From 1843 to the present time, the cases of aneurism treated by compression increase rapidly, owing mainly to a better theory of the mode of action of compressing instruments, and to a better practice founded on the theory. For this improvement—undoubtedly it may be so called—we are indebted to the Irish surgeons, and to none more than to our author. He was the first to set forth the true theory on which compression should be used, and has earnestly advocated this method of treatment on all proper occasions.

Dr. Bellingham has collected twenty-seven cases, which for the convenience of the reader and for reference we present in a tabular form, giving the name of the surgeon, the seat of the disease, and the number of days during which compression was employed:

| | | | | |
|---|---|---|---|---|
| 1. | Dr. Hutton - - - | Popliteal | Aneurism, - | 28 days. |
| 2. | Dr. Cusack - - - | “ | “ - | 31 “ |
| 3. | Dr. Bellingham - - | “ | “ - | 2 “ |
| 4. | Mr. Liston - - - | Femoral | “ - | 56 “ |
| 5. | Dr. Harrison - - | Popliteal | “ - | 93 “ |
| 6. | Mr. Liston - - - | Femoral | “ - | 30 “ |
| 7. | Dr. Bellingham - - | “ | “ - | 43 “ |
| 8. | Dr. Kirby - - - | Popliteal | “ - | 53 “ |
| 9. | Dr. Allan - - - | “ | “ - | 57 “ |
| 10. | Mr. Greatrex - - | “ | “ - | 21 “ |
| 11. | Dr. Cusack - - - | “ | “ - | 7 “ |
| 12. | Dr. Porter - - - | “ | “ - | 24 “ |
| 13. | Mr. Cusack - - - | “ | “ - | 20 “ |
| 14. | Mr. Porter - - - | “ | “ - | 20 “ |
| 15. | Mr. O'Ferrall, - - | “ | “ - | 33 “ |
| 16. | Mr. Jolley - - - | “ | “ - | 40 “ |
| 17. | Dr. McDonnell - - | “ | “ | not stated. |
| 18. | Mr. Dartnell - - | “ | “ - | 7 days. |
| 19. | Mr. Mackern - - | Femoral | “ - | 36 “ |
| 20. | Mr. Stork - - - | Popliteal | “ - | 91 “ |
| 21. | Mr. Stork - - - | “ | “ - | 22 “ |
| 22. | Mr. Cusack - - - | “ | “ - | 43 “ |
| 23. | Mr. Humfrey(?) - - | “ | “ - | 10 hours |
| 24. | Dr. Bellingham - - | “ | “ - | |
| 25. | ——— ——— - - | “ | “ - | |
| 26. | Mr. Armstrong - - | “ | “ - | 90 days. |
| 27. | Mr. Harrison - - | “ | “ - | |

In the 24th case, galvanism was tried in connection with pressure. The battery was applied once, but before a second application the patient died of erysipelas. The 25th case was treated at the hospital (St. Vincent's) to which the author is attached, but as it has not yet been published, he does not feel at liberty to give the details. In the 26th, pressure was made directly on the sac, as in the old method; the duration of compression was about 90 days. In the 27th, compression was discontinued after a fortnight, at the patient's request, and the vessel tied.

Dr. Bellingham devotes a chapter to the instruments employed in the treatment of these cases of aneurism. We have not room for the description of all of these, and therefore copy that of the one which he himself prefers, and which is called "*the clamp.*"

"The compressing instrument known at the surgical-instrument makers here under the name of the clamp, consists of an arc of steel, having at one extremity an oblong padded splint, at the other a nut containing a tourniquet screw connected with a smaller pad; the latter, although acted on by the screw, is so connected with it as to be readily turned in any direction. In using this instrument, the pad is laid on the artery, and the vessel is compressed by turning the screw, counter-pressure being made by the padded splint at the opposite side of the limb. It is constructed of different sizes so as to be adapted to compress the femoral artery as it crosses the horizontal ramus of the pubis, or the brachial artery low down in the arm.

"The clamp was first used as a compressing instrument in this city by a carpenter, the subject of popliteal aneurism, treated by Mr. Harrison (case 5). The pain occasioned by the apparatus originally in use induced the patient to try if the vessel could be compressed by an instrument called a clamp or cramp, which is used in his business for temporarily binding portions of wood-work until the glue intended to connect them becomes dry. It answered so well, and gave so little pain, that he continued to employ it in preference to any other until a cure was effected. The workmanship of the original instrument was, as may be supposed, rude, but it served as the model for those constructed since in this country.

"The clamp is a cheap and effective instrument, and not the least advantages which it possesses are that its construction is so simple that it is not liable to be put out of order; the principle upon which it acts is so plain as to be readily understood by the patient; and its application can be borne for a longer time and with less inconvenience than most other instruments."

Another means of compression was adopted by the author in case 7. The instrument in use got out of order, and, whilst it was undergoing repair, in order that the pressure might not be interrupted, a compress or pad was placed on the artery where it passes over the horizontal ramus of the pubis, and on this was laid a metal weight of seven pounds. In another case a weight of nine pounds was required to check the pulsation of the femoral artery. By this expedient the patient was relieved from the counter-pressure of the instrument, which in all cases is quite as irksome as the direct pressure. Indeed, the chief obstacle in the use of compression in aneurism, is the pain caused by the instruments. None of them can be borne long, if applied with a degree of force sufficient to diminish materially the current of blood in the artery. This led Dr. B. to employ two or three instead of one; these are placed upon the artery leading to the aneurismal sac, and when the pressure of one becomes painful it is relaxed, the other having been previously tightened; by thus alternating the pressure, compression can be constantly maintained for any length of time. This is an important improvement.

We have already stated that to our author belongs the credit of first advancing the true theory of compression. His views are contained in the following extract from the observations accompanying case 3, which was reported by him to the Surgical Society of Ireland in April, 1843.

"When it was considered absolutely necessary for the success of compression, that such an amount of pressure should be applied as was almost certain to produce sloughing of the part, and very certain to occasion intense pain and suffering; and when, in addition, this was to be prolonged through five successive nights and days, (as in the case reported by Mr. Guthrie, which I had quoted), we can readily understand why patients refused to submit to it, and we can easily account for the disrepute into which the practice fell, and for the unwillingness of surgeons to adopt this treatment, in preference to the simple operation of placing a ligature upon the femoral artery. It would, however, appear that it is not at all essential the circulation through the vessel leading to the aneurism should be completely checked, but rather the contrary: it may, perhaps, be advantageous at first, for a short period, by which the collateral circulation will be more certainly established; but the result of this case, if it does no more, establishes the fact, that *a partial current through an aneurismal sac will lead to the deposition of fibrin in its interior, and cause it within a few hours to be filled and obstructed, so as no longer to permit of the passage of blood through it.* Pressure, so as altogether to obstruct the circulation in an artery, must necessarily be slower in curing an aneurism, as it must, in some measure, act by causing obliteration of the vessel at the part to which the pressure has been applied; whereas *a partial current through the sac enables the fibrin to be readily entangled in the parietes of the sac in the first instance, and this goes on increasing until it becomes filled;* the collateral branches having been previously enlarged, the circulation is readily carried on through them."

The changes that take place in the aneurismal swelling after pressure has been applied for a short time to the artery, favor this theory. The tumor soon becomes hard and incompressible, and no longer collapses when the current of blood is completely cut off from it. The pathological appearances, in the few cases where an opportunity of observing them has occurred, also favor the author's views. In one (case 24) compression had been continued for several months; and the patient died of erysipelas, as already stated, thirteen days after the application of galvano-puncture, and before pulsation had ceased in the aneurism. The aneurismal sac was "almost filled by firm layers of fibrin, deposited in concentric laminæ, the layers next the sac being of a lighter color than those near the centre." The artery was healthy both above and below the sac, and was pervious through the whole popliteal space. In case 13, compression had been employed for about twenty days, and death took place forty-eight hours after cessation of pulsation in the aneurism: "the femoral artery was pervious to within the fourth of an inch of the sac; it was here filled up by a firm coagulum, which extended into the sac and completely filled it." In another case the patient had been treated by the author for popliteal aneurism on the right side (case 3), and subsequently for femoral aneurism in the opposite limb (case 7). He came under treatment a third time for aneurism of the aorta, of which he died two years and a half after the cure of the popliteal aneurism, and sixteen months after cure of the femoral aneurism. "At the site of each aneurismal sac the artery was converted into a solid, thick, flattened band, and its channel quite obliterated;" nothing remained of the original sacs, they having been absorbed along with the fibrin which had filled them; the femoral arteries of both limbs in their course down the thigh presented no alteration, and, as in the other cases, nothing indicated the points at which compression had been made.

The process of cure by compression would seem to be identical with that which nature herself now and then institutes. In nearly all cases of aneurismal tumor of some standing, there is more or less hardness from the deposition of solid matter in the interior; and on examination after spontaneous cure has taken place, the sac is found filled with fibrin arranged in concentric laminæ, the density and firmness of which diminish, whilst the color deepens, towards the centre. The separation of fibrin from fluent blood, is a familiar occurrence; as in the heart, when circulation through the chambers is impeded.

Again, the cure seems to be effected in the same way after the Hunterian operation. When a ligature is applied at a distance from the sac, the artery remains pervious below the ligature, and in a large majority of cases transmits a current of blood through the aneurismal tumor. Hence the frequent return of pulsation in the latter in a few hours or in a day or two after the operation. Solidification, however, soon commences; fibrin is deposited from the blood in its feeble passage through the sac, until at length the latter is filled. The ulterior changes in the tumor, after solidification, are of course the same whether cure has been effected by compression, by ligature, or spontaneously.

Dr. Bellingham considers at some length the circumstances which render this mode of treating aneurism tedious or the contrary. As it respects the *instrument*, the most essential points are that it should be simple in its constitution, and admit of ready application. The same instrument will scarcely be found to answer in two different cases. Theoretically, a pad or compress that will act on nothing but the artery, seems most correct; but practically a broad soft pad, which distributes the pressure over a large surface, is found to answer best. It is likewise important that the counter-pressure should be distributed over as large a surface as possible.

The *place* where compression is made requires consideration. In femoral and popliteal aneurism, the artery is easily compressed as it passes over the horizontal portion of the pubic bone, and also between Poupart's ligament and the junction of the saphena and femoral veins. Many patients bear pressure in the groin without inconvenience; but in others, the lymphatic glands become swollen and painful, or the limb benumbed and œdematous. A majority of the patients seen by the author preferred pressure lower down. No matter where pressure is applied, or how carefully it is graduated, if constantly maintained at the same point, it becomes painful, and must be relaxed. Hence the author's suggestion to employ two or three instruments in the manner we have heretofore mentioned.

No advantage is derived from *bandaging* the limb, at least in the earlier stages of the treatment; it interferes with the return of blood through the superficial veins, and with the increase of the collateral circulation. At a later period, when from the deposition of fibrin the sac becomes somewhat solid to the touch, a bandage and compress may be of service.

Much depends on the patient himself, and his disposition to co-operate with the surgeon. If he thinks lightly of his disease, or is unusually susceptible of pain, it is difficult to prevail on him to keep up compression. More frequently he finds the treatment irksome, and becomes dissatisfied, in consequence of the suffering caused by undue pressure in the beginning. On this point the author gives a caution:

"It is not necessary to repeat what I have already said respecting the degree of pressure which should be used; it will be sufficient to observe, that the compression at first ought always to be light; after a time, when tolerance of the remedy becomes established, we may increase it to the degree we consider necessary; but it need never be so great as to interrupt completely the circulation in the artery at the point upon which it is applied. Although it is advisable, in the early stage of the treatment, to maintain a certain degree of pressure night and day, yet in most instances it will be be found very difficult to do so; the patient cannot sleep unless the pressure is relaxed—if he does sleep, the pad becomes displaced, and the pressure is necessarily taken off the vessel. Indeed, if it were absolutely necessary to maintain permanent pressure in every instance until a cure was accomplished, we should seldom succeed; and that it is not necessary to do so, the details of the cases already given afford sufficient evidence, the pressure having been frequently intermitted and resumed according to circumstances. Once fibrin begins to be deposited in the sac the deposition will go on; although pressure may be very carelessly applied; and after a certain amount of fibrin has been deposited in the sac the instruments may be removed, and a cure will be accomplished nevertheless. Indeed it would appear from two cases previously quoted, that the patient may in some measure resume his ordinary habits and commence exercising the limb without interfering with the progress of the cure."

A number of circumstances connected with the sac, and its position with respect to the artery, also influence the duration of treatment. The most favorable case is where the disease is recent and the tumor still small. A large aneurism, that has increased slowly, generally contains solid matter, and is more speedily cured than a large one of recent formation with the contents still fluid. The author supposes that if the sac is situated on the anterior surface of the popliteal artery, it is not likely to attain a large size, owing to the unyielding nature of the surrounding parts; the entrance of blood into it is more or less impeded; and compression will probably cause a rapid deposition of fibrin. Or if fibrin to some extent has already been deposited, pressure by bandage and compress on the tumor itself may effect a speedy cure. When the aneurism grows from the posterior surface of the artery, it may attain a greater size, and be attended by severe pain from pressure on the nerve lying between it and the integuments. Compression of the artery in this case will be less speedy in its effects. Yet here also if from any cause fibrin comes to be deposited, direct pressure by a bandage and compress may induce rapid filling of the sac by forcing it down against the artery, or by diminishing the aperture between it and the artery. An aneurism springing from either side of the artery, is favorably placed for attaining a large size, and the treatment by compression either of the artery or sac would probably be more tedious.

It has already been mentioned that one of the objects of compression, as formerly practiced in popliteal aneurism, was to produce such enlargement of the collateral vessels as would insure sufficient circulation in the limb after ligation of the artery. But it has been ascertained that this increase of the collateral circulation is the very last thing that occurs; that it seldom commences until the disease is nearly cured; and that it is not completed until the aneurismal sac is filled with fibrin, and the artery obliterated. One of the most favorable signs, during the treatment of popliteal aneurism, is the pulsation of one of the articular arteries about the knee, as it shows that the circulation through the aneurism is permanently obstructed, and that nature is preparing new means to maintain the circulation in the limb.

Certain patients just before and after pulsation ceases in the tumor, whether this is effected spontaneously, or by compression, suffer severe pain about the knee. Dr. Bellingham supposes this is in some way connected with the dilatation of the anastomosing arteries.

In the last chapter, Dr. Bellingham proceeds to consider some of the advantages which compression has over the ligature. He thinks it simpler, safer, more certain, and more permanent.

"That compression effects the cure of aneurism by more *simple* means than the ligature is evidenced by the facts—1st. That the mode in which the consolidation of the aneurism is brought about by compression is exactly the same as that in which a natural or spontaneous cure occurs; and 2dly, because when a cure is effected by compression, the vessel is obliterated merely at the site of the aneurism; whereas when a ligature is applied in the usual situation at some distance from the tumor, the artery is obliterated both at the seat of the ligature and at the seat of the aneurism. Hence it is easy to understand why, when secondary hemorrhage followed the operation, the application of a second ligature higher up so seldom succeeded; and we can hardly be surprised at gangrene attacking a limb, the main artery of which is obliterated at three different points.

"That compression effects the cure of aneurism by *safer* means than the ligature is also evident, because its employment can be intermitted and resumed according to circumstances; and because no ill consequences have hitherto resulted from its use. On the other hand, the ligature of a large artery is always a precarious operation; when it is once applied, we must await its separation before the patient can be considered out of danger; and when it fails, which frequently happens, the case almost always terminates unfavorably, not from the increase of the disease, but from the operation performed for its relief. The artery in which aneurism (after the aorta) is most frequent, is the popliteal, and the ligature of the femoral artery for popliteal aneurism is more frequently unsuccessful than that of any other artery of equal size. Mr. Benjamin Phillips collected fifty-nine cases from various sources in which this vessel had been tied, in thirty-nine of which it failed; and although (as Mr. Storks observes) the acuracy of these statistics may be denied, "yet every surgeon must allow that the deligation of a main artery for aneurism is an operation (notwithstanding the successful results some practitioners can boast of,) attended with great risk." On the other hand, I have given a list of twenty-seven cases of aneurism treated by compression of the femoral artery, in twenty-five of which it succeeded perfectly; of the other two, one died of erysipelas before the cure was completed; the other was operated on at the patient's urgent request, and recovered. A mode of treatment therefore which is exempt from all risk has many advantages on the score of humanity, which alone ought to constitute a strong argument in its favor.

"The treatment of aneurism by compression is *more certain* than that by the ligature. We have already seen that the operation by ligature, however carefully performed, is a precarious one, and that it frequently fails; that secondary hemorrhage from ulceration of the artery at the site of the ligature or phlebitis not unfrequently follows it; or that suppuration of the sac, hemorrhage from it, or gangrene of the extremity, may ensue. Now, none of these unfortunate results have ever attended the treatment by compression, nor are any of them ever likely to follow it; because, in the first place, no injury whatever is inflicted upon either the artery or vein at the site of the pressure; and secondly, because the aneurismal sac, and the part of the artery from which it springs, are gradually filled up by fibrin, separated from the blood and deposited in the same way as when nature cures internal aneurism."

Experience thus far indicates that a cure once made by the new method is *permanent*, and the pathological facts formerly adduced show that it *must* be so, inasmuch as the sac is completely filled with fibrin. Secondary aneurism, or suppuration of the sac, never occurs after compression, though it often does after ligature.

Another advantage of compression is, that it can be adopted in the case of individuals who cannot be induced to submit to an operation. It may also be safely applied where the arterial coats are in such a state of disease that the ligature would necessarily lead to ulceration and secondary hemorrhage. Again, it may be instituted with every prospect of success where the aneurismal diathesis exists, where there is organic disease of the heart, and where the patient is of intemperate habits, or of broken down constitution; all circumstances which contra-indicate the Hunterian operation.

Having replied to some of the objections urged against compression. Dr. Bellingham says—

"I wish it to be understood that I do not advocate it as being free from inconvenience, free from trouble, or free from pain; the process by which compression effects the cure of aneurism is necessarily gradual, and requires time to be accomplished, and the surgeon, if he expects to succeed, must make up his mind to exercise a degree of patience which may be seldom called for in other cases; on the part of the patient likewise a considerable share of forbearance will be necessary; the former must be prepared to witness his exertions thwarted, and his endeavors fruitless for a long time; while the latter must be content to submit to confinement to bed for perhaps many consecutive weeks, and to the additional inconvenience of wearing a compressing apparatus during the greater part of that time. Although this is taking rather an unfavorable view of this method of treatment, and although in many of the cases which have been detailed, the cure was accomplished within a comparatively short period, yet it would be misleading those who have not seen this method employed, or who are about to try it for the first time, to let them suppose that it has no drawbacks; and that it does not occasionally prove both tedious and painful. Compression, however, possesses this advantage over the ligature that if persevered in, it cannot fail of effecting a cure; the cure may be impeded or protracted owing to a variety of causes, but from the manner in which the aneurismal sac becomes filled up, it is evident that every day will contribute a little, and every hour the pressure is applied something will be gained; and no matter how long the treatment may last, if the patient and surgeon have sufficient perseverance, a permanent cure will ultimately be accomplished, while the employment of the pressure does not involve the slightest risk."

Dr. Bellingham concludes the subject with the following summary:

"1. The arteries to which compression is applicable being far more frequently the subject of aneurism than those to which it is inapplicable, compression is calculated to supercede the ligature in the great majority of cases.

"2. The cure of aneurism by compression upon the artery between the aneurismal sac and the heart, according to the rules laid down here, is accomplished by the gradual deposition of the fibrin of the blood in the sac, until both the latter and the artery at the part are completely filled. The process is in fact exactly similar to that by which nature effects a spontaneous cure of aneurism.

"3. Such an amount of pressure as would cause inflammation and adhesion between the opposite sides of the artery at the point compressed is never required.

"4. The pressure should not be so great as to interrupt the circulation in the artery at the point compressed; an essential agent in the cure being that a current of blood should pass through the sac.

"5. Compression by means of two or more instruments, one of which is alternately relaxed, is much more effectual than by any single instrument, and in many instances the pressure can be maintained by the patient himself.

"6. The treatment of aneurism by compression does not involve the slightest risk to the patient, and if persevered in cannot fail of effecting a cure.

"7. A cure of aneurism effected by compression, according to the rules laid down here, must necessarily be permant; and in every case in which a cure has peen accomplished, the patients have remained well subsequently.

"8. The femoral artery remains pervious after the cure at the point at which the pressure had been applied, and no morbid change of any kind is to be detected either in the artery or vein at the site of the compression.

"9. When a cure is effected by compression, the vessel is obliterated only at the seat of the aneurism, and the artery at this part is eventually converted into an impervious ligamentous band.

"10. Compression effects the cure of aneurism by more simple and safer means than the ligature, while it is applicable to a number of cases in which the operation is contra-indicated or inadmissible.

"11. Compression is not necessarily a more tedious or more painful method of treating aneurism than the ligature, while it is much more certain, more likely to be permanent, and is free from all danger.

"12. Compression according to the rules laid down here, has little analogy with the old method which went by this name; and in fact has no greater resemblance to it than the Hunterian operation had to the operation for aneurism which it superseded."

Before finishing this paper, we must notice briefly the author's seventh chapter, in which he gives an account of the employment of certain chemical agents to produce coagulation of the blood in the aneurismal sac. We have deferred this until now, in order that our account of compression might not be interrupted.

Of the agents employed for this purpose, heat, galvanism, and actic acid are mentioned here. All of them possess the property of coagulating the blood out of the body. The first, suggested originally by Monteggia of Milan, received a trial at the hands of Sir Everard Home, in 1826. The case was one of femoral aneurism, and had been unsuccessfully treated by ligation on the distal side of the sac. An acupuncture needle having been introduced into the centre of the swelling, heat was applied by means of a spirit-lamp. This was repeated three times, with an interval of some days. Sometime after the last application, gangrene attacked the foot of the affected side, and, extending up the limb, soon caused the patient's death.

We find collected here seven cases that were treated by galvano-puncture, three of them successfully. In some of these it was employed along with compression. The mode of applying this remedy is to introduce two or more needles into the aneurismal tumor, so as to touch, or to cross each other at right angles, and then place them in communication with the poles of a galvanic pile. The needles ought previously to be covered with some insulating material, as asphaltum or sealing-wax. The author employed this means once (case 24) joined with compression. The case, already noticed by us in another connection, it will be recollected proved fatal from erysipelas. It should have been stated that the erysipelas began "in the middle of the thigh where the compressing instrument rested."

As to the mode in which galvanism forms a coagulum from the blood, the author, after quoting the views of Prof. Apjohn, observes—

"The coagulum developed by the action of the galvanic current upon blood would, therefore, appear to consist of *albumen*, derived from the decomposition of its serum; such a coagulum will be necessarily loose and flocculent, and altogether different from that which forms in an aneurismal sac under ordinary circumstances.

"In order that the albumen of the serum of the blood contained in an aneurismal sac may be coagulated, it would seem to be essential that the blood should be retained in it for a sufficient length of time to be acted on by the galvanic current; consequently, compression ought to be made upon the artery above and below the sac during the operation; if this precaution is not taken, the blood will pass through the sac too rapidly to permit of its decomposition.

"It appears to me that a coagulum might be developed from another source, when a galvanic current is conducted along needles introduced into an aneurismal sac: thus after the decomposition of the serum contained in the sac, and the separation of its albumen from the other constituents, it is not unreasonable to suppose that the fibrin and red globules of the same portion of blood (being then in some measure set free) might form a distinct coagulum; but of this we have no proof; and such a result could hardly be expected unless the galvanic current had been maintained for a sufficient length of time to coagulate completely the albumen of the serum contained in the sac. Even under these circumstances the coagulum would be loose and soft, and have little analogy with the fibrinous layers found in aneurism of some standing."

Galvanism may effect a cure of aneurism in two ways. 1. A small coagulum of albumen, formed as we have seen, may block up the opening into the artery on the distal side of the tumor; and if there is another coagulum formed (in the way the author supposes) of the fibrin and red globules, the chances of the artery's being obstructed will be greatly increased. At the same time compression must be made above and below the aneurism. 2. By exciting inflammation in the sac or neighboring parts, with effusion of serum and lymph, the swelling from which would produce compression of the artery. Of its value the estimate is not very high:

"On the whole it appears to me that much can never be expected from galvano-puncture as an agent in the cure of aneurism; it is doubtful if the current of a battery of moderate strength, however long continued, can develop a coagulum sufficiently firm to produce even the effects I have mentioned; and the employment of a powerful battery is not without risk if the aneurismal sac is of considerable size, or springs from a large artery. In addition, it is difficult to isolate completely the shaft of the acupuncture needles; the substance used for this purpose is either detached in the act of introducing them, or it is softened when the galvanic current is completed: hence the tissues through which the needles pass are acted on, and the strength of the current is necessarily wasted."

Wardrop proposed the injection of acetic acid into the sac, by means of Anel's syringe, the circulation through the vessel leading to the aneurism first to be arrested by means of compression. No trials of it have yet been reported.

Dr. Bellingham has expressed no opinion about any of these agents, except galvano-puncture. He places the full value upon this. The remaining two, in our humble judgment, are still less likely to be productive of any good. In using either of them, the surgeon must run the risk of exciting inflammation with all its wretched consequences. As to the acetic acid, despite the high authority of Wardrop, we are very much disposed to think that the man who should employ it where it was possible to employ any other means, ought to be punished for malpractice.

We here take leave of the author, with thanks for the instruction afforded in this volume. Much of its contents had reached us in fragments through other channels; but here we have these fragments in a connected history, constituting a well-timed and valuable contribution to surgical literature.

